# Supplementary material for: From sequence to enzyme mechanism using multi-label machine learning
Source: BMC Bioinformatics. 2014 May 19;15:150. doi: 10.1186/1471-2105-15-150 (PMC4229970; doi:10.1186/1471-2105-15-150)
Supplement: Additional file 2 — Java code of ml2db. Additional file ml2db_code.tar.gz contains the Java source code to run the multi-label machine learning experiments and save the results to database. The code’s Javadoc is included. [file 1471-2105-15-150-S2.zip › additional file 2/ml2db/utils/doc/uk/ac/standrews/utils/main/webutils/ClientHttpRequestUtils.html]

ClientHttpRequestUtils


JavaScript is disabled on your browser.


- Overview
- Package
- Class
- Use
- Tree
- Deprecated
- Index
- Help

- Prev Class
- Next Class

- Frames
- No Frames

- All Classes

- Summary:
- Nested |
- Field |
- Constr |
- Method

- Detail:
- Field |
- Constr |
- Method


uk.ac.standrews.utils.main.webutils

## Class ClientHttpRequestUtils

- java.lang.Object
- - uk.ac.standrews.utils.main.webutils.ClientHttpRequestUtils

- ---

    

  ```
  public class ClientHttpRequestUtils
  extends java.lang.Object
  ```

  Title: Client HTTP Request class

  Description: this class helps to send POST HTTP requests with various form
  data, including files. Cookies can be added to be included in the request.

  Version:
  :   1.0

  Author:
  :   Vlad Patryshev

- - ### Constructor Summary

    Constructors

    Constructor and Description

    `ClientHttpRequestUtils(java.lang.String urlString)`

    Creates a new multipart POST HTTP request for a specified URL string

    `ClientHttpRequestUtils(java.net.URL url)`

    Creates a new multipart POST HTTP request for a specified URL

    `ClientHttpRequestUtils(java.net.URLConnection connection)`

    Creates a new multipart POST HTTP request on a freshly opened
    URLConnection
  - ### Method Summary

    Methods

    | Modifier and Type | Method and Description |
    | --- | --- |
    | `java.io.InputStream` | `post()` posts the requests to the server, with all the cookies and parameters that were added |
    | `java.io.InputStream` | `post(java.util.Map parameters)` posts the requests to the server, with all the cookies and parameters that were added before (if any), and with parameters that are passed in the argument |
    | `java.io.InputStream` | `post(java.util.Map cookies, java.util.Map parameters)` posts the requests to the server, with all the cookies and parameters that were added before (if any), and with cookies and parameters that are passed in the arguments |
    | `java.io.InputStream` | `post(java.lang.Object[] parameters)` posts the requests to the server, with all the cookies and parameters that were added before (if any), and with parameters that are passed in the argument |
    | `java.io.InputStream` | `post(java.lang.String[] cookies, java.lang.Object[] parameters)` posts the requests to the server, with all the cookies and parameters that were added before (if any), and with cookies and parameters that are passed in the arguments |
    | `java.io.InputStream` | `post(java.lang.String name, java.lang.Object value)` post the POST request to the server, with the specified parameter |
    | `java.io.InputStream` | `post(java.lang.String name1, java.lang.Object value1, java.lang.String name2, java.lang.Object value2)` post the POST request to the server, with the specified parameters |
    | `java.io.InputStream` | `post(java.lang.String name1, java.lang.Object value1, java.lang.String name2, java.lang.Object value2, java.lang.String name3, java.lang.Object value3)` post the POST request to the server, with the specified parameters |
    | `java.io.InputStream` | `post(java.lang.String name1, java.lang.Object value1, java.lang.String name2, java.lang.Object value2, java.lang.String name3, java.lang.Object value3, java.lang.String name4, java.lang.Object value4)` post the POST request to the server, with the specified parameters |
    | `static java.io.InputStream` | `post(java.net.URL url, java.util.Map parameters)` posts a new request to specified URL, with parameters that are passed in the argument |
    | `static java.io.InputStream` | `post(java.net.URL url, java.util.Map cookies, java.util.Map parameters)` posts a new request to specified URL, with cookies and parameters that are passed in the argument |
    | `static java.io.InputStream` | `post(java.net.URL url, java.lang.Object[] parameters)` posts a new request to specified URL, with parameters that are passed in the argument |
    | `static java.io.InputStream` | `post(java.net.URL url, java.lang.String[] cookies, java.lang.Object[] parameters)` posts a new request to specified URL, with cookies and parameters that are passed in the argument |
    | `static java.io.InputStream` | `post(java.net.URL url, java.lang.String name1, java.lang.Object value1)` post the POST request specified URL, with the specified parameter |
    | `static java.io.InputStream` | `post(java.net.URL url, java.lang.String name1, java.lang.Object value1, java.lang.String name2, java.lang.Object value2)` post the POST request to specified URL, with the specified parameters |
    | `static java.io.InputStream` | `post(java.net.URL url, java.lang.String name1, java.lang.Object value1, java.lang.String name2, java.lang.Object value2, java.lang.String name3, java.lang.Object value3)` post the POST request to specified URL, with the specified parameters |
    | `static java.io.InputStream` | `post(java.net.URL url, java.lang.String name1, java.lang.Object value1, java.lang.String name2, java.lang.Object value2, java.lang.String name3, java.lang.Object value3, java.lang.String name4, java.lang.Object value4)` post the POST request to specified URL, with the specified parameters |
    | `void` | `setCookie(java.lang.String name, java.lang.String value)` adds a cookie to the requst |
    | `void` | `setCookies(java.util.Map cookies)` adds cookies to the request |
    | `void` | `setCookies(java.lang.String[] cookies)` adds cookies to the request |
    | `void` | `setParameter(java.lang.String name, java.io.File file)` adds a file parameter to the request |
    | `void` | `setParameter(java.lang.String name, java.lang.Object object)` adds a parameter to the request; if the parameter is a File, the file is uploaded, otherwise the string value of the parameter is passed in the request |
    | `void` | `setParameter(java.lang.String name, java.lang.String value)` adds a string parameter to the request |
    | `void` | `setParameter(java.lang.String name, java.lang.String filename, java.io.InputStream is)` adds a file parameter to the request |
    | `void` | `setParameters(java.util.Map parameters)` adds parameters to the request |
    | `void` | `setParameters(java.lang.Object[] parameters)` adds parameters to the request |

    - ### Methods inherited from class java.lang.Object

      `equals, getClass, hashCode, notify, notifyAll, toString, wait, wait, wait`

- - ### Constructor Detail


    - #### ClientHttpRequestUtils

      ```
      public ClientHttpRequestUtils(java.lang.String urlString)
                             throws java.io.IOException
      ```

      Creates a new multipart POST HTTP request for a specified URL string

      Parameters:
      :   `urlString` - the string representation of the URL to send request to

      Throws:
      :   `java.io.IOException`


    - #### ClientHttpRequestUtils

      ```
      public ClientHttpRequestUtils(java.net.URL url)
                             throws java.io.IOException
      ```

      Creates a new multipart POST HTTP request for a specified URL

      Parameters:
      :   `url` - the URL to send request to

      Throws:
      :   `java.io.IOException`


    - #### ClientHttpRequestUtils

      ```
      public ClientHttpRequestUtils(java.net.URLConnection connection)
                             throws java.io.IOException
      ```

      Creates a new multipart POST HTTP request on a freshly opened
      URLConnection

      Parameters:
      :   `connection` - an already open URL connection

      Throws:
      :   `java.io.IOException`
  - ### Method Detail


    - #### post

      ```
      public static java.io.InputStream post(java.net.URL url,
                             java.util.Map parameters)
                                      throws java.io.IOException
      ```

      posts a new request to specified URL, with parameters that are passed in
      the argument

      Parameters:
      :   `parameters` - request parameters

      Returns:
      :   input stream with the server response

      Throws:
      :   `java.io.IOException`

      See Also:
      :   `setParameters`


    - #### post

      ```
      public static java.io.InputStream post(java.net.URL url,
                             java.util.Map cookies,
                             java.util.Map parameters)
                                      throws java.io.IOException
      ```

      posts a new request to specified URL, with cookies and parameters that
      are passed in the argument

      Parameters:
      :   `cookies` - request cookies
      :   `parameters` - request parameters
      :   `url` - a URL

      Returns:
      :   a stream

      Throws:
      :   `java.io.IOException`


    - #### post

      ```
      public static java.io.InputStream post(java.net.URL url,
                             java.lang.Object[] parameters)
                                      throws java.io.IOException
      ```

      posts a new request to specified URL, with parameters that are passed in
      the argument

      Parameters:
      :   `parameters` - request parameters

      Returns:
      :   input stream with the server response

      Throws:
      :   `java.io.IOException`

      See Also:
      :   `setParameters`


    - #### post

      ```
      public static java.io.InputStream post(java.net.URL url,
                             java.lang.String name1,
                             java.lang.Object value1)
                                      throws java.io.IOException
      ```

      post the POST request specified URL, with the specified parameter

      Parameters:
      :   `name` - parameter name
      :   `value` - parameter value

      Returns:
      :   input stream with the server response

      Throws:
      :   `java.io.IOException`

      See Also:
      :   `setParameter`


    - #### post

      ```
      public static java.io.InputStream post(java.net.URL url,
                             java.lang.String name1,
                             java.lang.Object value1,
                             java.lang.String name2,
                             java.lang.Object value2)
                                      throws java.io.IOException
      ```

      post the POST request to specified URL, with the specified parameters

      Parameters:
      :   `name1` - first parameter name
      :   `value1` - first parameter value
      :   `name2` - second parameter name
      :   `value2` - second parameter value

      Returns:
      :   input stream with the server response

      Throws:
      :   `java.io.IOException`

      See Also:
      :   `setParameter`


    - #### post

      ```
      public static java.io.InputStream post(java.net.URL url,
                             java.lang.String name1,
                             java.lang.Object value1,
                             java.lang.String name2,
                             java.lang.Object value2,
                             java.lang.String name3,
                             java.lang.Object value3)
                                      throws java.io.IOException
      ```

      post the POST request to specified URL, with the specified parameters

      Parameters:
      :   `name1` - first parameter name
      :   `value1` - first parameter value
      :   `name2` - second parameter name
      :   `value2` - second parameter value
      :   `name3` - third parameter name
      :   `value3` - third parameter value

      Returns:
      :   input stream with the server response

      Throws:
      :   `java.io.IOException`

      See Also:
      :   `setParameter`


    - #### post

      ```
      public static java.io.InputStream post(java.net.URL url,
                             java.lang.String name1,
                             java.lang.Object value1,
                             java.lang.String name2,
                             java.lang.Object value2,
                             java.lang.String name3,
                             java.lang.Object value3,
                             java.lang.String name4,
                             java.lang.Object value4)
                                      throws java.io.IOException
      ```

      post the POST request to specified URL, with the specified parameters

      Parameters:
      :   `name1` - first parameter name
      :   `value1` - first parameter value
      :   `name2` - second parameter name
      :   `value2` - second parameter value
      :   `name3` - third parameter name
      :   `value3` - third parameter value
      :   `name4` - fourth parameter name
      :   `value4` - fourth parameter value

      Returns:
      :   input stream with the server response

      Throws:
      :   `java.io.IOException`

      See Also:
      :   `setParameter`


    - #### post

      ```
      public static java.io.InputStream post(java.net.URL url,
                             java.lang.String[] cookies,
                             java.lang.Object[] parameters)
                                      throws java.io.IOException
      ```

      posts a new request to specified URL, with cookies and parameters that
      are passed in the argument

      Parameters:
      :   `cookies` - request cookies
      :   `parameters` - request parameters

      Returns:
      :   input stream with the server response

      Throws:
      :   `java.io.IOException`

      See Also:
      :   `setCookies`,
          `setParameters`


    - #### post

      ```
      public java.io.InputStream post()
                               throws java.io.IOException
      ```

      posts the requests to the server, with all the cookies and parameters
      that were added

      Returns:
      :   input stream with the server response

      Throws:
      :   `java.io.IOException`


    - #### post

      ```
      public java.io.InputStream post(java.util.Map parameters)
                               throws java.io.IOException
      ```

      posts the requests to the server, with all the cookies and parameters
      that were added before (if any), and with parameters that are passed in
      the argument

      Parameters:
      :   `parameters` - request parameters

      Returns:
      :   input stream with the server response

      Throws:
      :   `java.io.IOException`

      See Also:
      :   `setParameters`


    - #### post

      ```
      public java.io.InputStream post(java.util.Map cookies,
                             java.util.Map parameters)
                               throws java.io.IOException
      ```

      posts the requests to the server, with all the cookies and parameters
      that were added before (if any), and with cookies and parameters that are
      passed in the arguments

      Parameters:
      :   `cookies` - request cookies
      :   `parameters` - request parameters

      Returns:
      :   input stream with the server response

      Throws:
      :   `java.io.IOException`

      See Also:
      :   `setParameters`,
          `setCookies`


    - #### post

      ```
      public java.io.InputStream post(java.lang.Object[] parameters)
                               throws java.io.IOException
      ```

      posts the requests to the server, with all the cookies and parameters
      that were added before (if any), and with parameters that are passed in
      the argument

      Parameters:
      :   `parameters` - request parameters

      Returns:
      :   input stream with the server response

      Throws:
      :   `java.io.IOException`

      See Also:
      :   `setParameters`


    - #### post

      ```
      public java.io.InputStream post(java.lang.String name,
                             java.lang.Object value)
                               throws java.io.IOException
      ```

      post the POST request to the server, with the specified parameter

      Parameters:
      :   `name` - parameter name
      :   `value` - parameter value

      Returns:
      :   input stream with the server response

      Throws:
      :   `java.io.IOException`

      See Also:
      :   `setParameter`


    - #### post

      ```
      public java.io.InputStream post(java.lang.String name1,
                             java.lang.Object value1,
                             java.lang.String name2,
                             java.lang.Object value2)
                               throws java.io.IOException
      ```

      post the POST request to the server, with the specified parameters

      Parameters:
      :   `name1` - first parameter name
      :   `value1` - first parameter value
      :   `name2` - second parameter name
      :   `value2` - second parameter value

      Returns:
      :   input stream with the server response

      Throws:
      :   `java.io.IOException`

      See Also:
      :   `setParameter`


    - #### post

      ```
      public java.io.InputStream post(java.lang.String name1,
                             java.lang.Object value1,
                             java.lang.String name2,
                             java.lang.Object value2,
                             java.lang.String name3,
                             java.lang.Object value3)
                               throws java.io.IOException
      ```

      post the POST request to the server, with the specified parameters

      Parameters:
      :   `name1` - first parameter name
      :   `value1` - first parameter value
      :   `name2` - second parameter name
      :   `value2` - second parameter value
      :   `name3` - third parameter name
      :   `value3` - third parameter value

      Returns:
      :   input stream with the server response

      Throws:
      :   `java.io.IOException`

      See Also:
      :   `setParameter`


    - #### post

      ```
      public java.io.InputStream post(java.lang.String name1,
                             java.lang.Object value1,
                             java.lang.String name2,
                             java.lang.Object value2,
                             java.lang.String name3,
                             java.lang.Object value3,
                             java.lang.String name4,
                             java.lang.Object value4)
                               throws java.io.IOException
      ```

      post the POST request to the server, with the specified parameters

      Parameters:
      :   `name1` - first parameter name
      :   `value1` - first parameter value
      :   `name2` - second parameter name
      :   `value2` - second parameter value
      :   `name3` - third parameter name
      :   `value3` - third parameter value
      :   `name4` - fourth parameter name
      :   `value4` - fourth parameter value

      Returns:
      :   input stream with the server response

      Throws:
      :   `java.io.IOException`

      See Also:
      :   `setParameter`


    - #### post

      ```
      public java.io.InputStream post(java.lang.String[] cookies,
                             java.lang.Object[] parameters)
                               throws java.io.IOException
      ```

      posts the requests to the server, with all the cookies and parameters
      that were added before (if any), and with cookies and parameters that are
      passed in the arguments

      Parameters:
      :   `cookies` - request cookies
      :   `parameters` - request parameters

      Returns:
      :   input stream with the server response

      Throws:
      :   `java.io.IOException`

      See Also:
      :   `setParameters`,
          `setCookies`


    - #### setCookie

      ```
      public void setCookie(java.lang.String name,
                   java.lang.String value)
                     throws java.io.IOException
      ```

      adds a cookie to the requst

      Parameters:
      :   `name` - cookie name
      :   `value` - cookie value

      Throws:
      :   `java.io.IOException`


    - #### setCookies

      ```
      public void setCookies(java.util.Map cookies)
                      throws java.io.IOException
      ```

      adds cookies to the request

      Parameters:
      :   `cookies` - the cookie "name-to-value" map

      Throws:
      :   `java.io.IOException`


    - #### setCookies

      ```
      public void setCookies(java.lang.String[] cookies)
                      throws java.io.IOException
      ```

      adds cookies to the request

      Parameters:
      :   `cookies` - array of cookie names and values (cookies[2\*i] is a name,
          cookies[2\*i + 1] is a value)

      Throws:
      :   `java.io.IOException`


    - #### setParameter

      ```
      public void setParameter(java.lang.String name,
                      java.io.File file)
                        throws java.io.IOException
      ```

      adds a file parameter to the request

      Parameters:
      :   `name` - parameter name
      :   `file` - the file to upload

      Throws:
      :   `java.io.IOException`


    - #### setParameter

      ```
      public void setParameter(java.lang.String name,
                      java.lang.Object object)
                        throws java.io.IOException
      ```

      adds a parameter to the request; if the parameter is a File, the file is
      uploaded, otherwise the string value of the parameter is passed in the
      request

      Parameters:
      :   `name` - parameter name
      :   `object` - parameter value, a File or anything else that can be
          stringified

      Throws:
      :   `java.io.IOException`


    - #### setParameter

      ```
      public void setParameter(java.lang.String name,
                      java.lang.String value)
                        throws java.io.IOException
      ```

      adds a string parameter to the request

      Parameters:
      :   `name` - parameter name
      :   `value` - parameter value

      Throws:
      :   `java.io.IOException`


    - #### setParameter

      ```
      public void setParameter(java.lang.String name,
                      java.lang.String filename,
                      java.io.InputStream is)
                        throws java.io.IOException
      ```

      adds a file parameter to the request

      Parameters:
      :   `name` - parameter name
      :   `filename` - the name of the file
      :   `is` - input stream to read the contents of the file from

      Throws:
      :   `java.io.IOException`


    - #### setParameters

      ```
      public void setParameters(java.util.Map parameters)
                         throws java.io.IOException
      ```

      adds parameters to the request

      Parameters:
      :   `parameters` - "name-to-value" map of parameters; if a value is a file, the
          file is uploaded, otherwise it is stringified and sent in the
          request

      Throws:
      :   `java.io.IOException`


    - #### setParameters

      ```
      public void setParameters(java.lang.Object[] parameters)
                         throws java.io.IOException
      ```

      adds parameters to the request

      Parameters:
      :   `parameters` - array of parameter names and values (parameters[2\*i] is a
          name, parameters[2\*i + 1] is a value); if a value is a file,
          the file is uploaded, otherwise it is stringified and sent in
          the request

      Throws:
      :   `java.io.IOException`


- Overview
- Package
- Class
- Use
- Tree
- Deprecated
- Index
- Help

- Prev Class
- Next Class

- Frames
- No Frames

- All Classes

- Summary:
- Nested |
- Field |
- Constr |
- Method

- Detail:
- Field |
- Constr |
- Method
